# Identification of pyroptosis‐related lncRNAs for constructing a prognostic model and their correlation with immune infiltration in breast cancer

**DOI:** 10.1111/jcmm.16969

**Published:** 2021-10-10

**Authors:** Wenchang Lv, Yufang Tan, Chongru Zhao, Yichen Wang, Min Wu, Yiping Wu, Yuping Ren, Qi Zhang

**Affiliations:** ^1^ Department of Plastic and Cosmetic Surgery Tongji Hospital Tongji Medical College Huazhong University of Science and Technology Wuhan China

**Keywords:** breast cancer, lncRNA, prognosis, pyroptosis, risk model

## Abstract

The inflammasome‐dependent cell death, which is denoted as pyroptosis, might be abnormally regulated during oncogenesis and tumour progression. Long non‐coding RNAs (LncRNAs) are pivotal orchestrators in breast cancer (BC), which have the potential to be a biomarker for BC diagnosis and therapy. The present study aims to explore the correlation between pyroptosis‐related lncRNAs and BC prognosis. In this study, a profile of 8 differentially expressed lncRNAs was screened in the TCGA database and used to construct a prognostic model. The BC patients were divided into high‐ and low‐risk groups dependent on the median cutoff of the risk score in the model. Interestingly, the risk model significantly distinguished the clinical characteristics of BC patients between high‐ and low‐risk groups. Then, the risk score of the model was identified to be an excellent independent prognostic factor. Notably, the GO, KEGG, GSEA and ssGSEA analyses revealed the different immune statuses between the high‐ and low‐risk groups. Particularly, the 8 lncRNAs expressed differentially in BC tissues between two risk subgroups in vitro validation. Collectively, this constructed well‐validated model is of high effectiveness to predict the prognosis of BC, which will provide novel means that is applicable for BC prognosis recognition.

## 1 INTRODUCTION

Breast cancer (BC) is the most frequently diagnosed female malignancy and one of the leading causes of cancer‐related mortality among women worldwide.^1^ BC is a highly complex cancer type with histological and molecular heterogeneity, posing an enormous threat to women health due to its extremely high recurrence rate and mortality rate.^2^ Although the therapeutic efficacy of BC gains momentum dramatically, unfortunately, the lack of effective hallmarks and diagnostic tools to predict prognosis or long‐term survival in BC patients remains a major obstacle to improving strategies for BC detection and treatment.^3^ Therefore, it is imperative to explore novel therapeutic targets and reliable prognostic models for optimal clinical outcomes in BC.

Pyroptosis is a form of cell death, which is known as inflammasome‐dependent programmed cell death (PCD).^4^ When persistent inflammation came about, initial activation and assembly of inflammasome complexes occurred in the host cell, followed by further activation of caspase and production of proinflammatory cytokines, finally leading to pyroptotic cell death.^5^ It has been proposed that pyroptosis‐related genes directly participate in tumour development. For example, gasdermin superfamily proteins are the executors of pyroptosis, the N‐terminal of which oligomerized to form pore across the cell membrane, leading to the secretion of IL‐1β and IL‐18.^6^ Similarly, An et al. found that the growth and metastasis of triple‐negative breast cancer (TNBC) cells could be suppressed by tetraarsenic in the pyroptotic cell death manner, which was activated via the reactive oxygen species (ROS)‐mediated caspase‐3/gasdermin E pathway.^7^ Furthermore, the caspase‐1–mediated inflammasome pathway involving in NLRP1‐, NLRP3‐, NLRC4‐, AIM2‐ and pyrin‐inflammasome is known as canonical inflammasome pathway in pyroptosis.^8^ It was worth noting that the tumour growth and metastasis in BC animal model were correlated with the activation of the inflammasome and elevated IL‐1β expression at primary and metastatic sites.^9^

Long non‐coding RNAs (LncRNAs), more than 200 nucleotides in length, are RNA transcripts with low coding capability.^10^ Functionally, lncRNAs can regulate gene expression at multiple levels, including transcription, chromatin organization, RNA processing and translation.^11^ LncRNAs mainly serve as scaffolds or decoys to recruit or isolate effector proteins from corresponding DNA, RNA or protein targets.^12^ Notably, lncRNA expression is of higher cell and tissue specificity compared to mRNAs.^13^ Numerous studies have verified that tumour‐related lncRNAs could alter the intrinsic properties of tumour cells to remodel TME.^14^ Moreover, the dysregulation of lncRNAs was associated with the clinical stage and prognosis of several tumours, including prostate cancer, lung cancer and BC.^10^ For example, Niu et al. found that lncRNA RAB11B‐AS1 enhanced the expression of angiogenic factors including VEGFA and ANGPTL4 in hypoxic BC cells by increasing recruitment of RNA polymerase II, thus promoting BC angiogenesis and migration.^15^ Meanwhile, in the study of Liang et al., lncRNA BCRT1 was significantly up‐regulated in BC tissues, which was correlated with BC poor prognosis.^16^

Interestingly, with the development of bioinformatics, plenty of studies reported the signature construction based on ncRNAs to predict BC prognosis. Early in 2014, Zhou et al. sequenced 14 miRNAs to establish a miRNA‐signature acting as a prognostic marker in estrogen receptor (ER)‐positive BC.^17^ In 2020, Tang et al. also manufactured a signature consisting of 8 lncRNAs for ER‐BC‐positive analysis, which could predict survival in patients receiving endocrine therapy.^18^ Thus, it could conclude that ncRNAs, represented by lncRNAs, had the immeasurable potential of prognostic value and ability in BC. More importantly, Ye et al. constructed a prognostic signature composed of 7 pyroptosis‐related mRNAs in 2021.^19^ This study mainly aimed to explore these genes in ovarian cancer, but the predictive potential of pyroptosis‐related lncRNAs in BC and their association with immune state were not clearly deciphered. These results supported the feasibility of the risk model constructed with pyroptosis‐related genes in tumour prognosis. Unfortunately, few studies have reported the prognostic value of pyroptosis‐related lncRNAs in BC in recent years.

**FIGURE 1:**
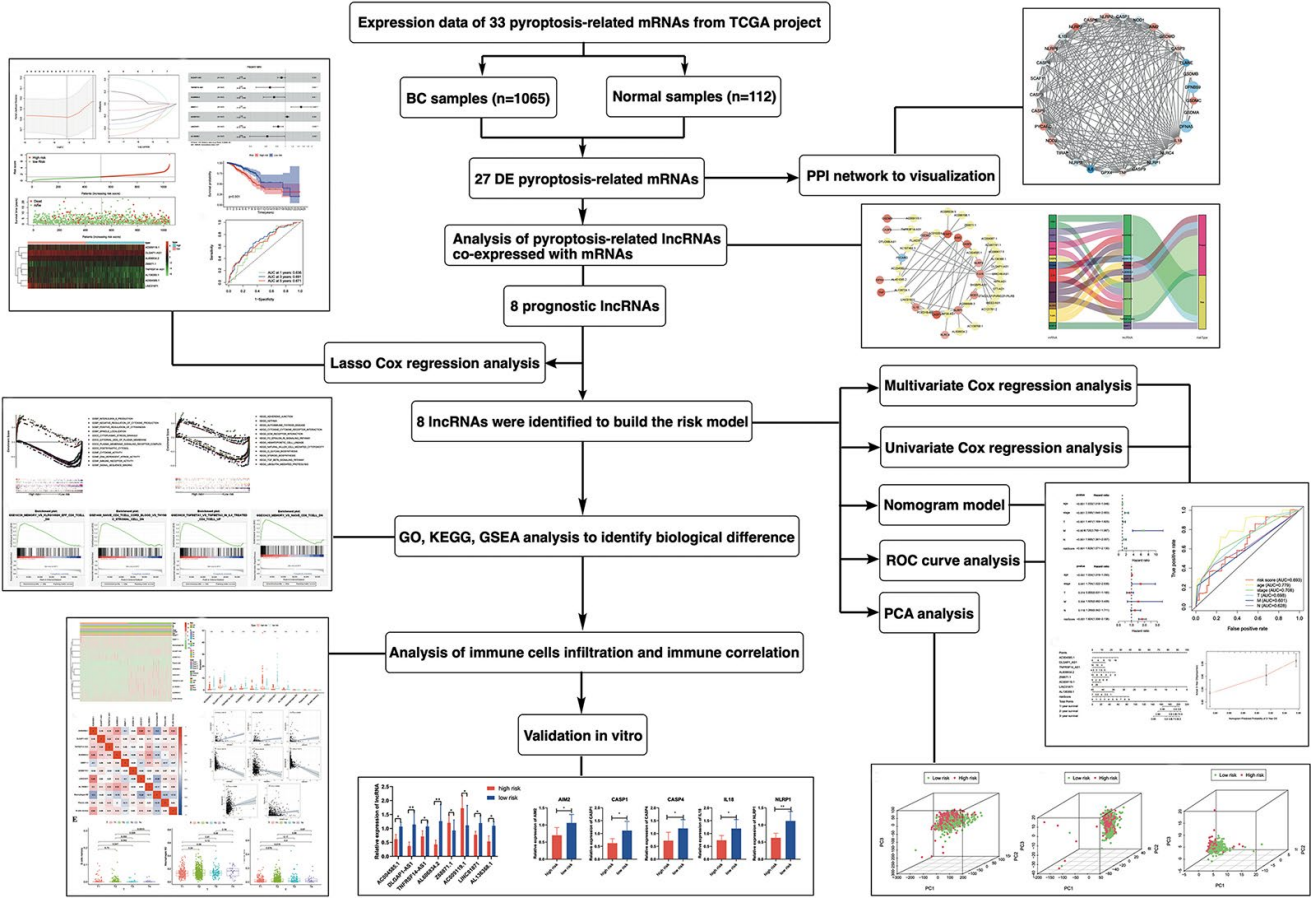
The flowchart of this study

Given the expression profile and biological effects of pyroptosis and lncRNAs in BC, the present study aims to explore the correlation between pyroptosis‐related lncRNAs and BC prognosis. Initially, we screened and selected a profile of 8 differentially expressed (DE) lncRNAs analysed from the TCGA database and accordingly constructed a prognostic‐predicting risk model. The BC patients were divided into high‐risk and low‐risk groups dependent on the median cutoff of risk score based on this model. Then, the efficacy of the prognosis value of this model was evaluated by analyses of univariate and multivariate Cox regression, nomogram model, ROC curve, and principal component analysis (PCA), and the biological differences in the two groups were validated by gene ontology (GO), kyoto encyclopedia of genes and genomes (KEGG) and gene set enrichment analysis (GSEA) analysis. The 8 pyroptosis‐lncRNAs associated with the immune state were also confirmed by correlation analyses and in vitro assay. The detailed flowchart could be seen in Figure 1. To the end, the findings of this study will provide a theoretical reference for the development of a high‐efficiency prognostic assessment tool for combating BC.

## 2 METHODS

### 2.1 Data collection and processing

The RNA sequencing data of pyroptosis‐related genes and the corresponding clinical features of 1,065 BC patients were downloaded from the TCGA data portal (https://portal.gdc.cancer.gov/), for the subsequent difference and co‐expression analysis. Patients without survival information were excluded for further evaluation. The prognostic values and clinical characteristics of lncRNAs and immune cells were also verified in TCGA databases.

### 2.2 Identification of DE genes

Based on previous reports,^19^ a total of 33 pyroptosis‐related mRNAs were extracted for identification, and then 27 pyroptosis‐related mRNAs between 1,065 BC patients and 112 normal samples were finally confirmed by Mann–Whitney–Wilcoxon test according to the available mRNA expression data from the TCGA. The “limma” package was used to identify pyroptosis‐related DE genes with a *p* value <0.05. The DE genes were notated as follows: * if *p* < 0.05, ** if *p* < 0.01 and *** if *p* < 0.001. The protein‐protein interaction (PPI) network of the DE genes was constructed with a Search Tool for the Retrieval of Interacting Genes (STRING) (http://string‐db.org/) and the threshold combined score was set as ≥0.4. Besides, the Cytoscape software (version 3.7.1) was used to visualize the PPI network.

### 2.3 Acquisition of pyroptosis‐related lncRNAs related to BC prognosis

Pyroptosis‐related lncRNAs were obtained through co‐expression analysis with genes in the TCGA datasets. The prognostic pyroptosis‐related lncRNAs were further selected, by taking the intersection of lncRNAs associated with the prognosis of BC patients via Cytoscape and Sankey diagram.

### 2.4 Construction and validation of the prognostic risk model based on the pyroptosis‐related lncRNAs

Lasso Cox regression analysis was performed to identify the relationship between prognostic signatures of pyroptosis‐related lncRNAs and BC risk. Then, about 8 pyroptosis‐related genes were screened out to construct the optimal prognostic model by using the “glmnet” software package. Importantly, the risk score was calculated using the following formula: the risk score = Expression_mRNA1_ × Coefficient_mRNA1_ + Expression_mRNA2_ × Coefficient_mRNA2_ +... + Expression_mRNAn_ × Coefficient_mRNAn_. Furthermore, the BC patients were divided into high‐risk and low‐risk groups using the median cutoff of risk score based on the risk model. Notably, the prognostic factors were distinguished to be positive or negative via the hazard ratio (HR) from univariate and multivariate Cox regression analyses. The gene with HR >1 was considered to be a risk gene, and the gene with HR <1 was considered to be a protective one.

### 2.5 The survival and the receiver operating characteristic (ROC) analysis

The survival analysis was conducted by R language v4.0.2 with package survival and survminer, and the survival differences between different groups were identified via the Kaplan–Meier survival analysis. The sensitivity and specificity of the predictive model on BC overall survival (OS) at 1‐, 3‐ and 5‐year were evaluated by the ROC curve, which was plotted by package time ROC of R language v4.0.2. Subsequently, the package survival ROC was used to analyse all the independent risk factors of different clinical characteristics, including age, gender, clinical stage, TNM stage and risk score, for the goal of predicting 3‐year OS in BC patients.

### 2.6 The construction of nomogram model and PCA

In the TCGA datasets, the risk score and 8 pyroptosis‐related lncRNAs were included to build a nomogram using the “rms” R package to predict the 1‐, 2‐ and 3‐year survival rate of BC patients. The calibration curve was utilized to estimate the discrimination and accuracy of the nomogram. The PCA was utilized to cluster the BC patients based on the expression patterns of pyroptosis‐related genes. Moreover, the distribution of all the patients was visualized via a 3D scatterplot.

### 2.7 The GO, KEGG, GSEA and ssGSEA enrichment analysis

The GO (http://www.geneontology.org/) and the KEGG (http://www.genome.jp/kegg/) pathway analysis were used to explore the differences of potential biological function and signaling pathway between the two risk groups. The GSEA (http://software.broadinstitute.org/gsea/index.jsp) was performed to explore the potential differences of immune‐related pathways between two risk groups. The cut‐off criteria were set as nominal *p* < 0.01. The “gsva” R package was utilized to conduct the single‐sample gene set enrichment analysis (ssGSEA) to evaluate the infiltrating scores of 16 immune cells and the activities of 13 immune‐related pathways.

### 2.8 Quantitative real‐time polymerase chain reaction (qRT‐PCR) analysis

The total RNA was extracted by using the TRIzol reagent kit (Invitrogen) and the RNA concentration was detected by a K5800 spectrophotometer (Kaiao). The complementary DNA (cDNA) was synthesized by using the PrimeScript RT kit (Takara) at 103°C for 5 s, 37°C for 10 min and 4°C for 15 min. Next, the qRT‐PCR analysis was performed using a SYBR Green PCR master mix (Yeasen) in a QuantStudio1 PCR (ABI Q1, USA) at the following settings: 95°C for 10 min, followed by 40 cycles of 95°C for 15 s and 60°C for 1 min. The primer sequences for 8 lncRNAs and 5 mRNAs detection were displayed in detail in Table S1 and Table S2, respectively. All the gene expression levels were collected and quantified using the 2^−△△Ct^ method. Three independent experiments were operated.

### 2.9 Statistics analysis

All statistical analyses were performed by using R version 4.0.5 and GraphPad Prism (version 8.0). The single‐factor analysis of variance was used to compare the expression differences of the pyroptosis‐related genes between BC and normal tissues. The Kaplan–Meier method was used to compare the OS between subgroups. The prognostic value was estimated by univariate and multivariate Cox proportional hazards regression analyses. The Mann–Whitney test was used to compare the immune cell infiltration and immune pathway activation between the two groups. The correlation between two variables was evaluated by Pearson correlation analyses. The values of *p* < 0.05 were considered to be significant.

## 3 RESULTS

### 3.1 Identification of DE pyroptosis‐related mRNAs in normal and BC tissues

**FIGURE 2:**
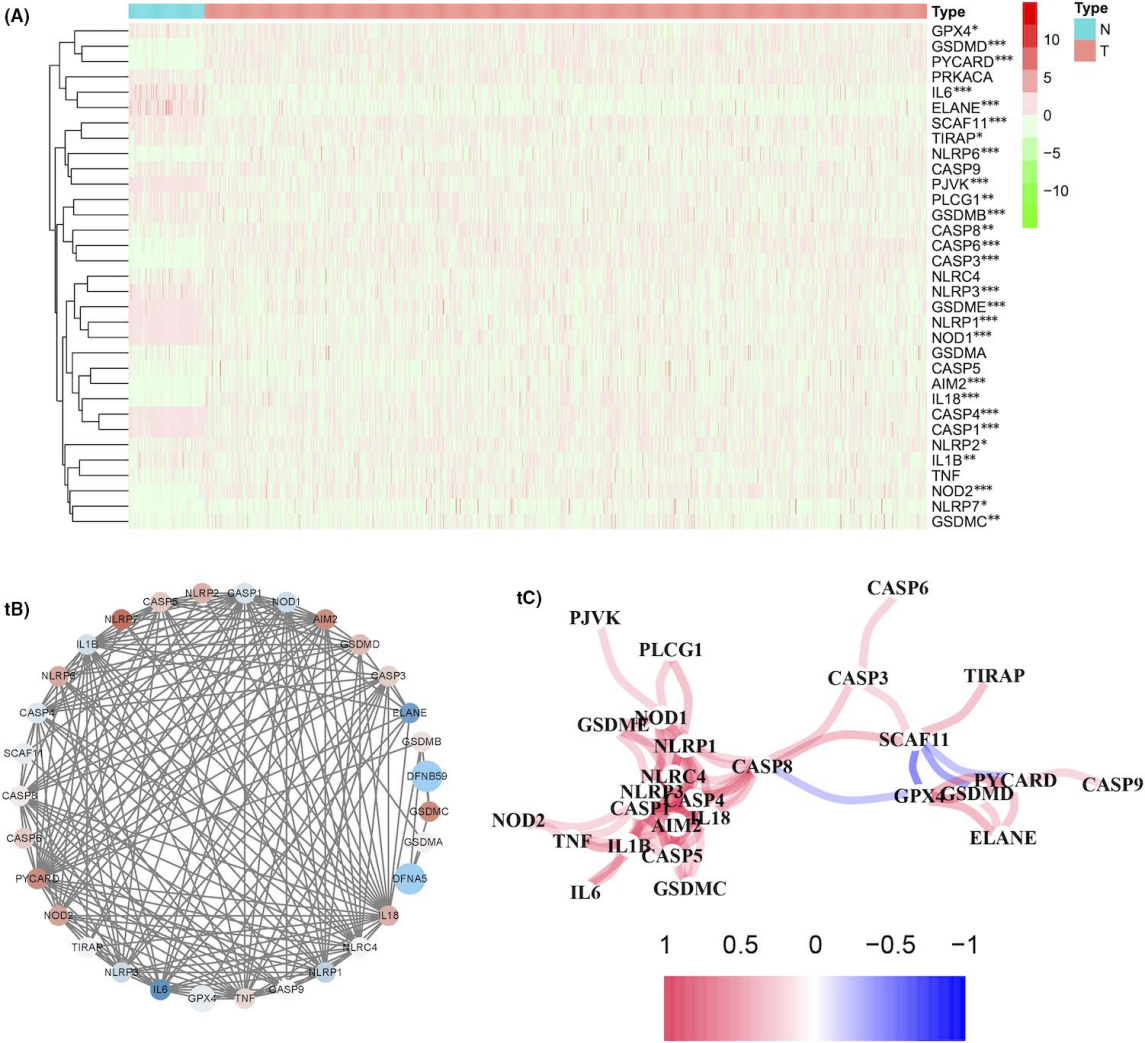
The expression features of pyroptosis‐related mRNAs and the interaction network. (A) A heatmap of the expression level of 33 pyroptosis‐related mRNAs between the normal (N, blue) and BC tissues (T, red). Green: down‐regulation; Red: up‐regulation; *p < 0.05, **p < 0.01; ***p < 0.001. (B) PPI network visualizing the interactions of the pyroptosis‐related mRNAs (interaction score =0.7). The different colours represented different expressions in BC. Red: up‐regulation; Blue: down‐regulation. The deeper the colours, the greater the expressed degree. (C) The correlation network of the pyroptosis‐related mRNAs. Red line: positive correlation; Blue line: negative correlation. The deeper the colours, the stronger the relevance

By comparing the expression levels of 33 pyroptosis‐correlated mRNAs in the TCGA dataset from 112 normal and 1,065 BC tissues, about 27 mRNAs were finally identified with statistically DE patterns (*p* < 0.05) (Figure 2A). Among them, a total of 14 mRNAs were downregulated significantly while the other 13 mRNAs were upregulated in the BC group (Figure 2A). Subsequently, the PPI was constructed to visualize the interactions among 31 pyroptosis‐related genes (Figure 2B). Besides, the results of Figure 2C also showed the correlation network between all DE mRNAs in another manner, both confirming the specific interactive patterns of intense complexity between pyroptosis‐related mRNAs.

### 3.2 Co‐expression network of pyroptosis‐related mRNAs and lncRNAs with prognostic value

**FIGURE 3:**
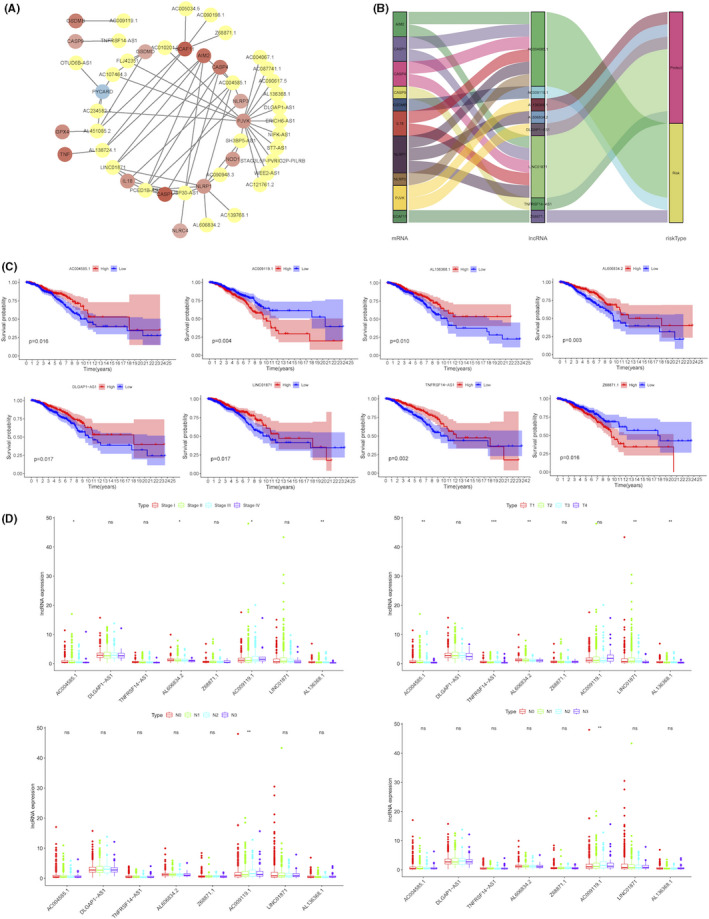
Identification of pyroptosis‐related lncRNAs with significant prognostic value and clinical characteristics in BC. (A) Cytoscape constructing a co‐expression network of pyroptosis‐related lncRNAs‐mRNAs. Yellow represented lncRNAs. Red with different depth represented mRNAs. (B) Sankey diagram visualizing a co‐expression network of 8 pyroptosis‐related lncRNAs and mRNAs with prognostic value in BC. (C) The Kaplan–Meier survival analysis of the 8 pyroptosis‐related lncRNAs in BC. (D) The correlation of the expression of the 8 pyroptosis‐related lncRNAs with BC clinicopathological factors. ns: no significance; *p < 0.05, **p < 0.01; ***p < 0.001

The pyroptosis‐related lncRNAs were further enrolled based on the co‐expression pattern with mRNAs to construct a co‐expression network, which consisted of 16 pyroptosis‐correlated mRNAs and 31 lncRNAs (Figure 3A). Then, after taking the intersection of the lncRNAs significantly associated with the prognosis of BC patients, 8 pyroptosis‐related lncRNAs were selected. Among them, the Sankey diagram visualized the 6 protective lncRNAs (AC004585.1, AL136368.1, AL606834.2, DLGAP1‐AS1, LINC01871, TNFRSF14‐AS1) and the 2 risk lncRNAs (AC009119.1 and Z68871.1) (Figure 3B). Moreover, the results from Pearson correlation analysis about the prognostic value of the above 8 lncRNAs demonstrated that BC patients with high expression of AC009119.1 and Z68871.1 were prone to have a shorter survival time (*p* < 0.05) (Figure 3C). Importantly, compared with the TNM stage, the expression of the pyroptosis‐related lncRNAs was significantly related to the clinical stage of BC patients (*p* < 0.05) (Figure 3D).

### 3.3 Construction of risk model based on pyroptosis‐correlated lncRNAs

**FIGURE 4:**
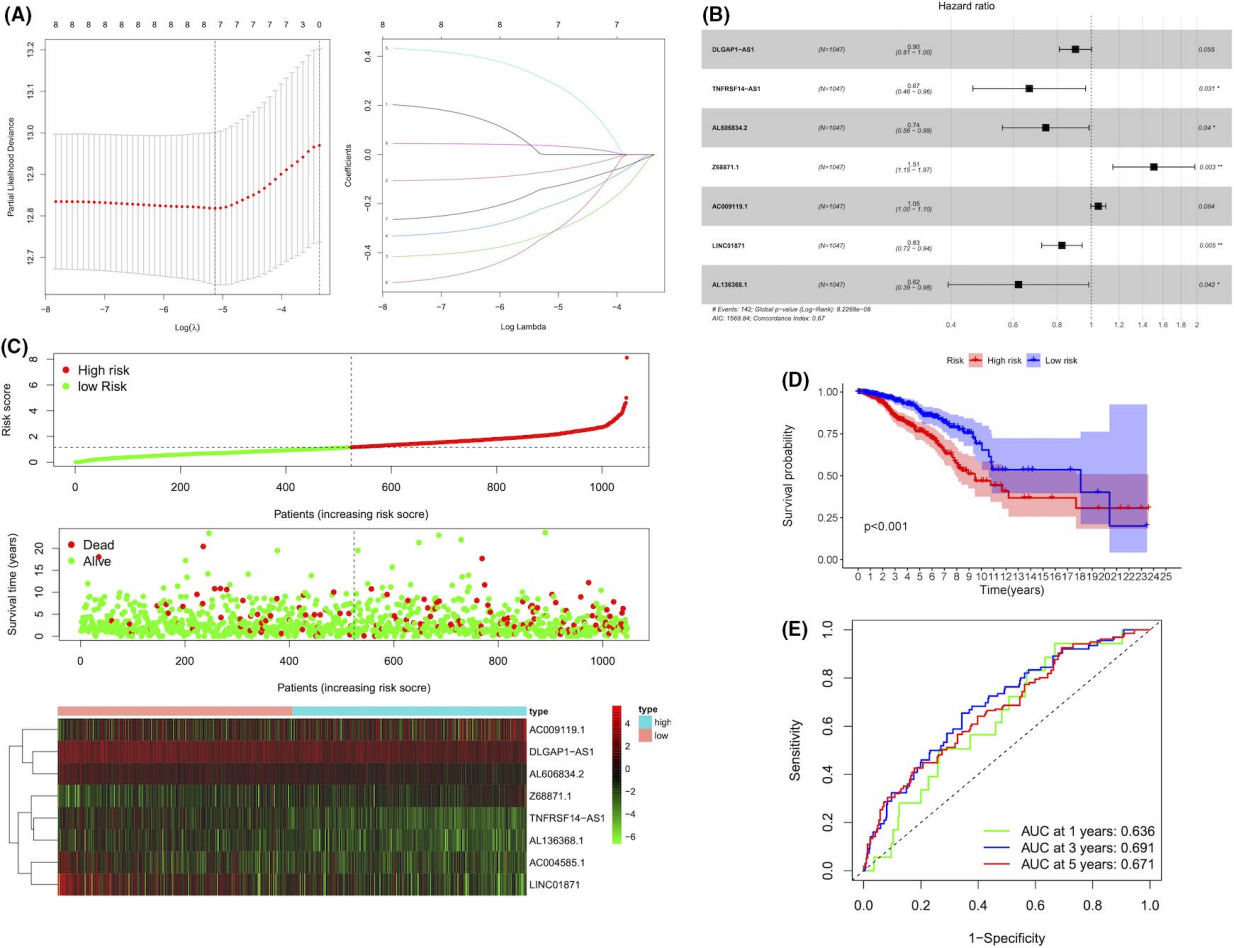
Construction of a predictive risk model in BC. (A) LASSO regression of the 8 prognostic pyroptosis‐related lncRNAs. (B) The forest plot of the HR for the correlation between 8 pyroptosis‐related lncRNAs and OS of BC patients. (C) The distribution and survival status of BC patients with different risk scores. The green and red dots represented survival and death, respectively. The heatmap exhibited the expression levels of 8 pyroptosis‐related lncRNAs in the high‐ and low‐risk groups. (D) The Kaplan–Meier survival analysis showing the survival time of BC patients between high‐ and low‐risk groups. (E) The ROC curves of the risk model at 1‐, 3‐ and 5‐year

Importantly, the above identified 8 lncRNAs by Lasso Cox regression analysis on 33 pyroptosis‐related lncRNAs were successfully used to build the prognostic risk model (Figure 4A). The forest plot showed the corresponding HRs and 95% CIs of the 8 lncRNAs, demonstrating that Z68871.1 was the risk factor of BC prognosis, (Figure 4B), which was consistent with the Kaplan–Meier analysis in Figure 2C. Furthermore, the coefficients of 8 lncRNAs in this risk model were utilized to calculate the risk score. The risk score =AC009119.1 × (0.045182) + DLGAP1‐AS1 × (−0.110273) + AL606834.2 × (−0.342492) + Z68871.1 × (0.442614) + TNFRSF14‐AS1 × (−0.424380) + AL136368.1 × (−0.342492) + AC004585.1 × (0.219343) + LINC01871 × (−0.277494). Then, we divided the 1,047 BC patients into low‐risk (524 patients) and high‐risk groups (523 patients) based on the median threshold of risk score. Interestingly, it was found that the high‐risk groups possessed significantly worse OS than the low‐risk groups (*p* < 0.05) (Figure 4C). Likewise, the death probability of BC patients with the high‐risk score was significantly higher than those with low‐risk score by the Kaplan–Meier curve (median time =2.101 years vs. 2.524 years, *p* < 0.001) (Figure 4D). Moreover, with the increase of risk score, the expression levels of AC009119.1 and Z68871.1 were increased, whereas the expression levels of the other 6 lncRNAs were decreased (*p* < 0.05) (Figure 4C). In addition, the ROC curve showed that the risk score had significant predictive sensitivity and specificity, with the area under curve (AUC) of 0.636, 0.691 and 0.671 in 1‐, 3‐ and 5‐year, respectively (Figure 4E).

### 3.4 The prognostic value of the risk model

**FIGURE 5:**
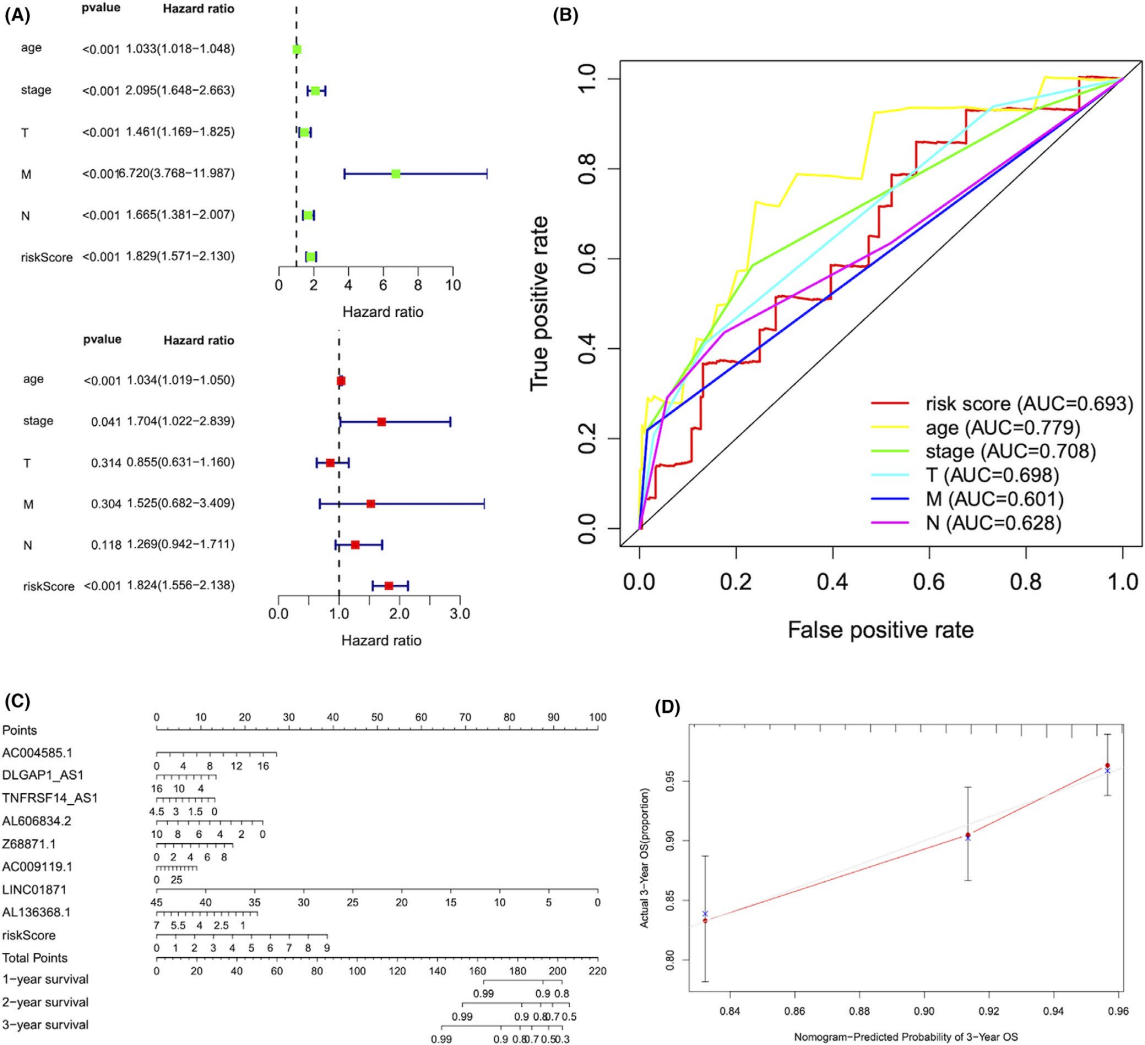
The prognostic value of the risk score. (A) The univariate and multivariate Cox regression analysis of the risk score and other clinical feature prognostic value. (B) The AUC for the risk score and other clinical features (age, stage and TNM stage) based on the ROC curves. (C) Construction of a nomogram containing 8 pyroptosis‐related lncRNAs and the risk score to predict 1‐, 2‐ and 3‐year OS of BC. (D) Calibration plots of the nomograms in terms of the agreement between nomogram‐predicted and observed 3‐year survival outcomes of BC. The 45° dashed line represented the ideal observation. The red line represented the actual prediction of the model

Notably, the risk score was identified as an independent predictor of survival in BC patients, by the analysis of univariate Cox regression (HR =1.829, 95% CI: 1.571–2.130) and multivariate Cox regression (HR =1.824, 95% CI: 1.556–2.138) combined with other clinical characteristics (Figure 5A). Meanwhile, the risk score was equipped with independent and splendid predictive ability compared with other factors with an AUC of 0.779, which was elucidated by the ROC curve analysis (Figure 5B). To quantitatively predict the probability of clinical OS in BC patients, a prognostic nomogram model using risk scores was established (Figure 5C). The calibration plot for the predictive probability of 3‐year OS by nomogram exhibited excellent consistency with actual observation (Figure 5D). Hence, these results suggested both the risk model and the nomogram model had good discrimination and accuracy in predicting the OS of BC patients.

### 3.5 The functional analyses and PCA based on the risk model

**FIGURE 6:**
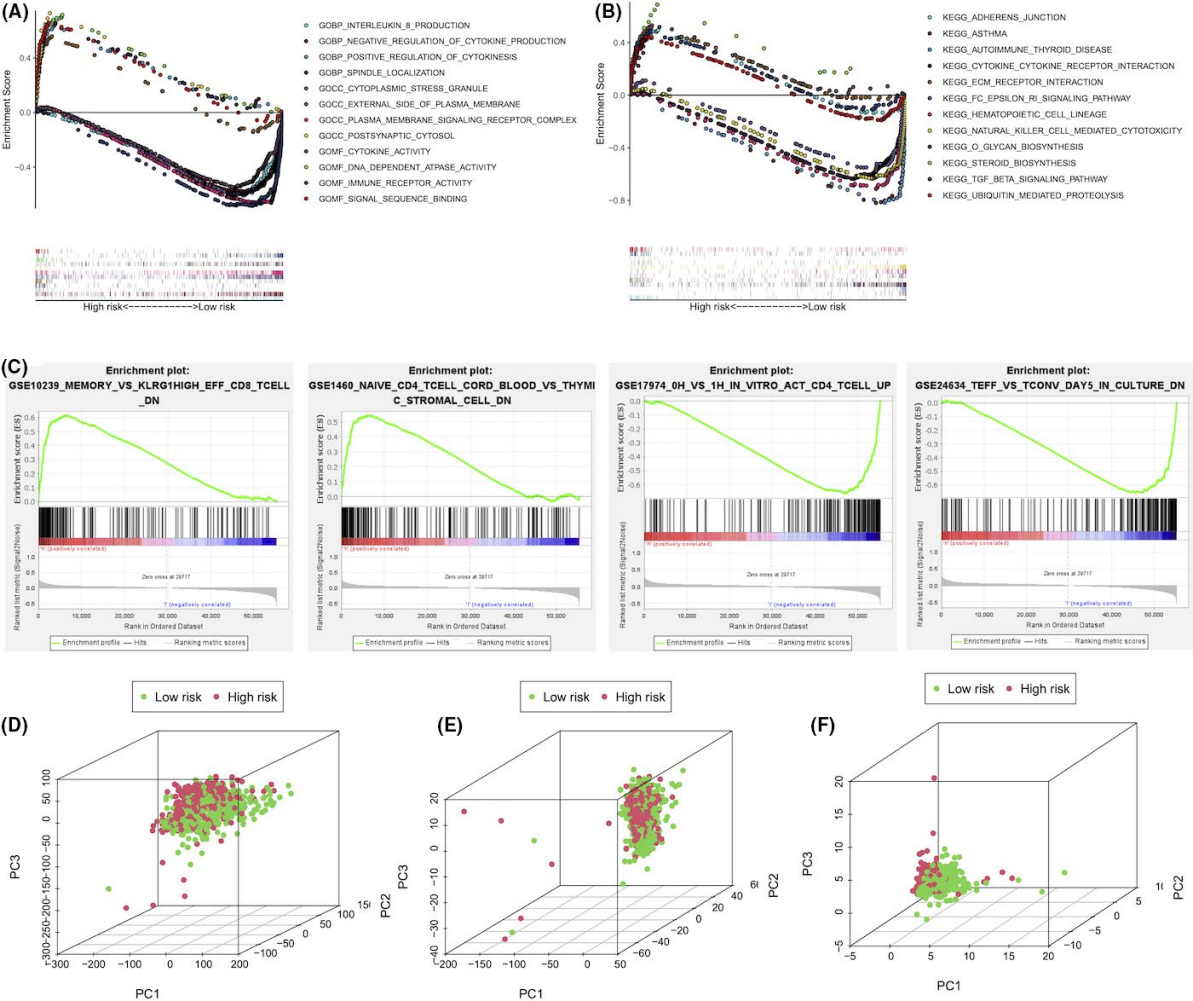
Patients with high‐ and low‐risk scores had different pyroptotic and oncogenic statuses. The GO (A), KEGG (B) and GSEA (C) analysis between high‐ and low‐risk groups based on the risk model in BC. The 3D scatterplot of the PCA showing the distribution of BC patients based on all pyroptosis‐related genes (D), all pyroptosis‐related lncRNAs (E) and 8 pyroptosis‐related lncRNAs in our risk model (F)

The results from the GO and KEGG analysis showed differentially biological function between the high‐ and low‐risk groups (Figure 6A). The intracellular biogenesis primarily occurred in the high‐risk group in comparison to the low‐risk group, including positive regulation of cytokinesis, spindle localization, cytoplasmic stress granule, postsynaptic cytosol, DNA‐dependent ATPase activity and signal sequence binding (Figure 6A). Further KEGG analysis showed that high‐risk groups also enriched in several pathways and processes, which were closely related to tumour progression, including adhesion junctions, ECM receptor interactions, O‐glycan biosynthesis, steroid biosynthesis, TGF‐β signaling pathways and ubiquitin‐mediated proteolysis (Figure 6B). Notably, the GSEA results revealed that immune‐related pathways were differentially enriched in high‐ and low‐risk groups of BC patients (Figure 6C). Additionally, the PCA was performed to overview different distribution patterns between the two risk groups. It was confirmed that compared with the expression patterns of all the pyroptosis‐correlated mRNAs (Figure 6D) and all the pyroptosis‐correlated lncRNAs (Figure 6E), our risk model based on the 8 pyroptosis‐correlated lncRNAs had the optimal ability to divide the high‐ and low‐risk groups of BC patients into different subgroups clearly (Figure 6F).

### 3.6 The different immune activity between high‐ and low‐risk subgroups

**FIGURE 7:**
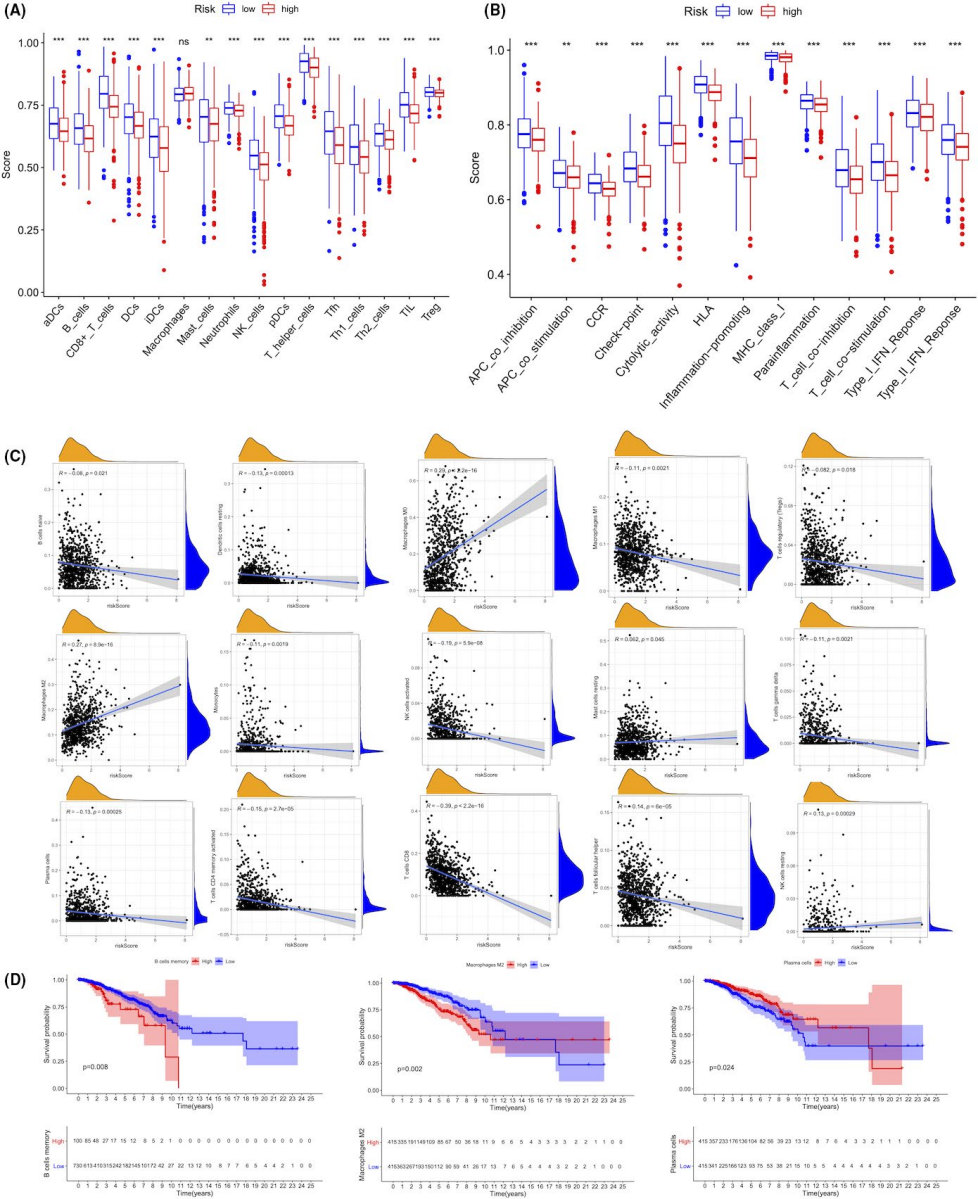
Patients with high‐ and low‐risk scores had different immune statuses. Comparison of the ssGSEA scores of 16 types of immune cells (A) and 13 immune‐related pathways (B) between low‐ (blue box) and high‐risk (red box) groups in the TCGA project. (C) The correlation between the risk model and immune cells infiltration. (D) The Kaplan–Meier survival analysis of the immune cell infiltration in BC patients

The ssGSEA analysis in the TCGA project was used to analyse the 16 different immune cell types between high‐ and low‐risk groups, finding that the infiltration levels of the 15 immune‐related cell types except macrophages were significantly different between the two subgroups (*p* < 0.05) (Figure 7A). Besides, the high‐risk group generally had lower infiltration levels of immune cells, especially CD8+ T cells, neutrophils, natural killer (NK) cells, T helper (Th) cells (Tfh, Th1, and Th2 cells), regulatory T (Treg) cells and tumour‐infiltrating lymphocytes (TILs) (*p* < 0.05) (Figure 7A). Meanwhile, the ssGSEA analysis revealed that in the TCGA project, the activities of all the 13 immune‐related pathways were significantly lower in the high‐risk group than in the low‐risk group (*p* < 0.05) (Figure 7B). It was noted that about 15 infiltrating immune cells were identified to be significantly associated with risk score (*p* < 0.05) (Figure 7C). The infiltration of immune cells significantly affected the prognosis of BC patients, and the higher levels of memory B cells and M2 macrophages predicted the worse survival prognosis (*p* < 0.05) (Figure 7D). Therefore, the above results highlighted the immune‐regulating role of the pyroptosis‐related lncRNAs between high‐ and low‐risk subgroups.

### 3.7 Correlation of pyroptosis‐correlated lncRNAs with immune state

**FIGURE 8:**
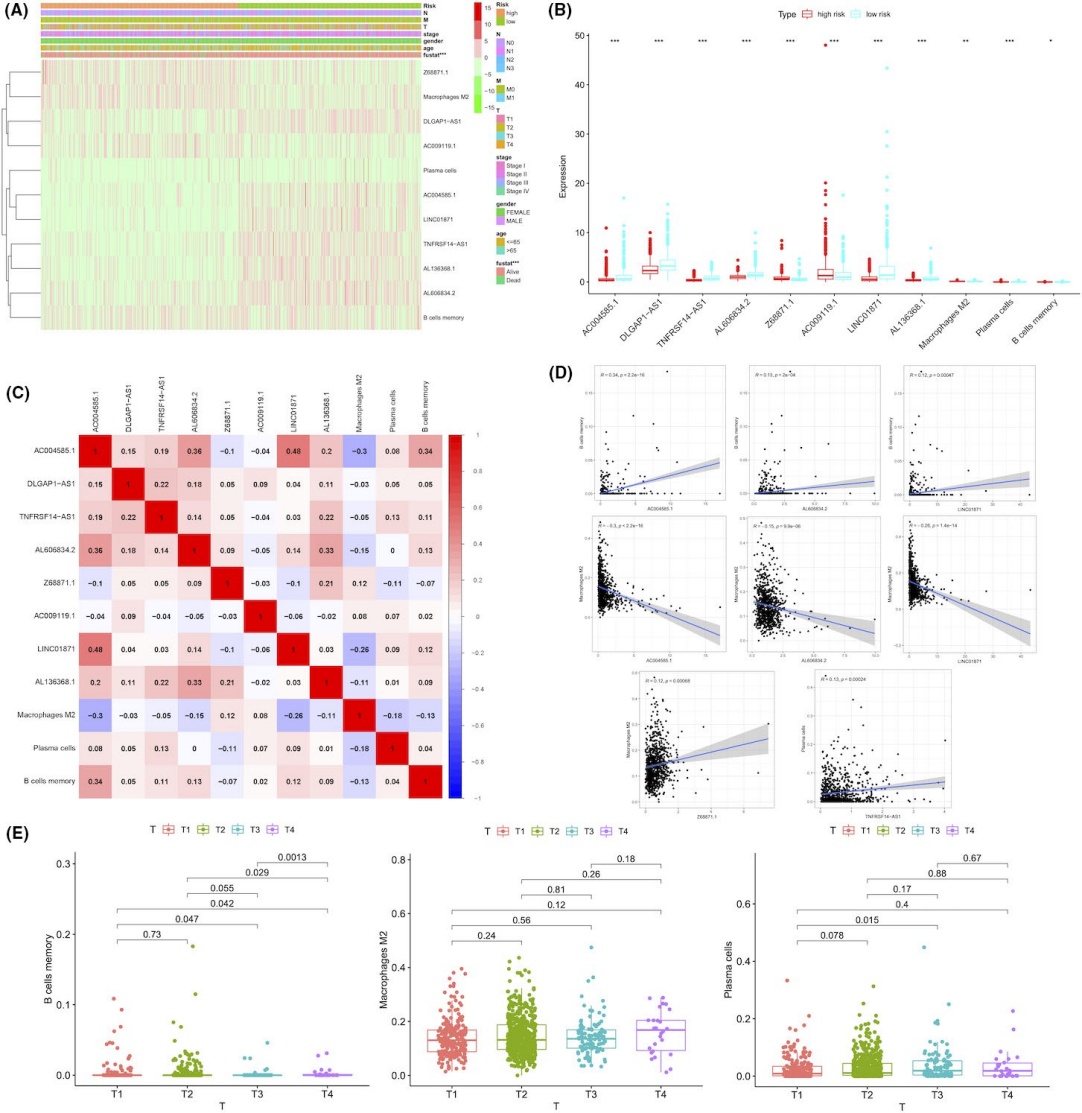
The 8 pyroptosis‐related lncRNAs were related to immune infiltration and BC clinical features. (A) A heatmap visualizing the distribution of the 8 pyroptosis‐related lncRNAs and 3 immune cells in two risk groups and different BC clinical outcomes (age, gender, TNM stage and state). (B) The expression of 8 pyroptosis‐related lncRNAs and 3 immune cells between high‐ and low‐risk groups. *p < 0.05, **p < 0.01; ***p < 0.001. (C–D) The correlation of the 8 lncRNAs with 3 immune cells. (E) The correlation of the 3 immune cells with T stage of BC patients

The heatmap from the TCGA project visualized the clinical features of BC patients, including age, gender, clinical stage, TNM stage and the survival status, as well as the abundance of 3 immune cells (memory B cells, M2 macrophages and plasma cells) (Figure 8A). The distribution of survival status (survival or death), which was a close predictor of prognosis in BC patients, was significantly different between the high‐risk and low‐risk groups (*p* < 0.05) (Figure 8A). The tumour‐immunoreactive components that influenced the prognosis of BC, including memory B cells and M2 macrophages, were significantly higher in the high‐risk group than that in the low‐risk group (*p* < 0.05) (Figure 8B). In addition, the Pearson correlation analysis was conducted for analysing the correlation between 8 lncRNAs related to pyroptosis and 3 immune cells. The result strongly implied that memory B cell infiltration was significantly positively correlated with 3 lncRNAs (AC004585.1, AL606834.2 and LINC01871), and that plasma cell infiltration was positively correlated with TNFRSF14‐AS1 (Figure 8C–D). Besides, the M2 macrophage infiltration was significantly negatively correlated with 3 lncRNAs (AC004585.1, LINC01871, AL606834.2) and positively correlated with Z68871.1 (*p* < 0.05). Interestingly, the memory B cell infiltration was significantly associated with the clinical stage in BC patients (*p* < 0.05) (Figure 8E).

### 3.8 The validation of pyroptosis‐related genes in BC cells in vitro

**FIGURE 9:**
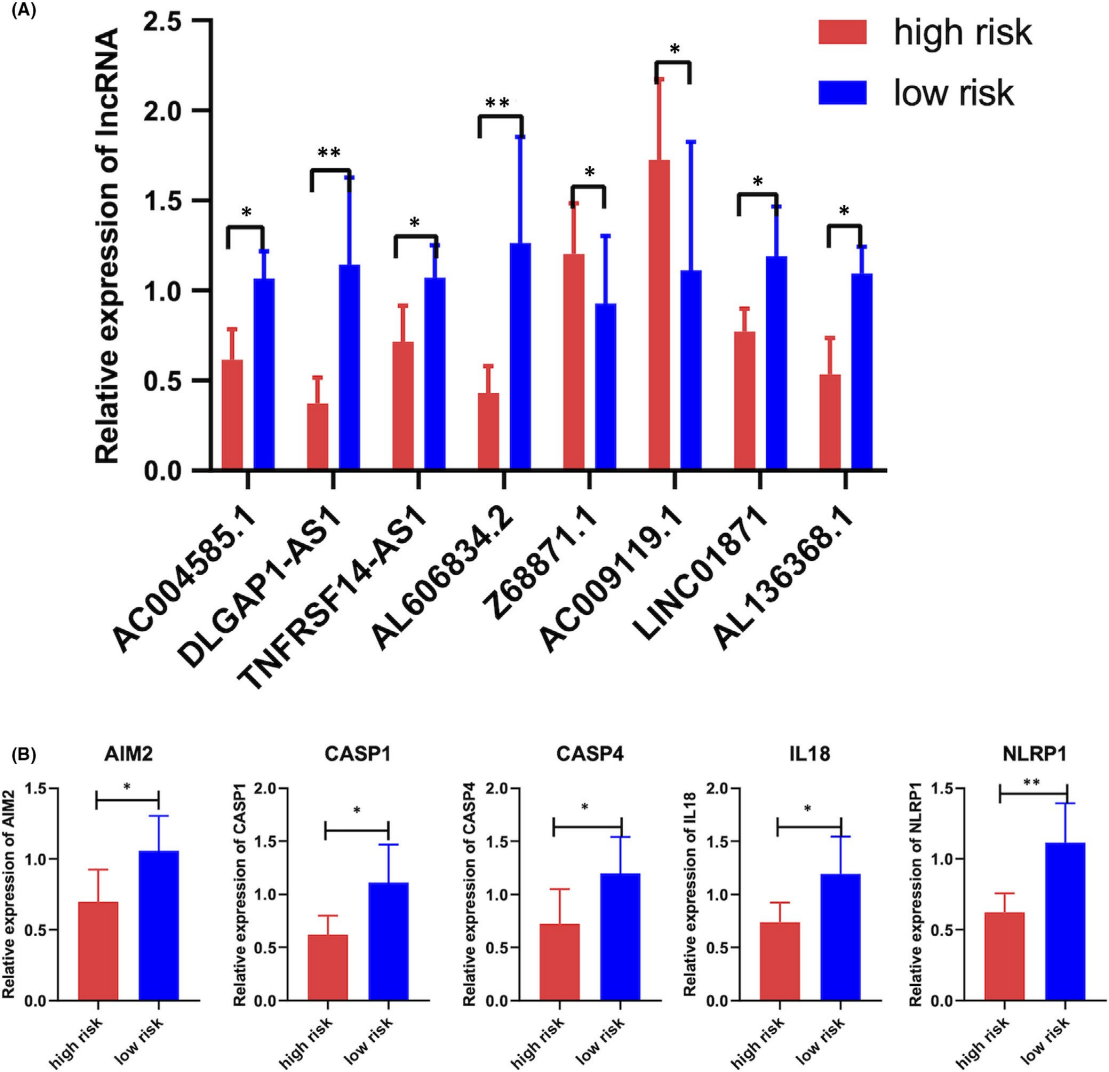
Validation of the pyroptosis‐related genes expressed differentially in high‐ and low‐risk groups in vitro. The expression level of 8 pyroptosis‐related lncRNAs (A) and 5 pyroptosis‐related mRNAs (B) in the high‐ and low‐risk group detected by qRT‐PCR. *p < 0.05, **p < 0.01

Ultimately, the qRT‐PCR was performed to validate the expression characteristics of the 8 lncRNAs in our risk model. The results showed that all those 8 lncRNAs expressed differentially in BC tissues between two risk subgroups (Figure 9A). Especially, AC009119.1 and Z68871.1 were elevated and other 6 lncRNAs were declined in the high‐risk group (Figure 9A). Furthermore, several pyroptosis‐related mRNAs, including AIM2, CASP1, CASP4, IL18 and NLRP1, were mainly enriched in the low‐risk group (Figure 9B). Notably, these mRNAs were co‐expressed with AC004585.1, AL606834.2 and LINC01871, which were significantly correlated with memory B cells infiltration.

## 4 DISCUSSION

In our study, a risk model based on 8 pyroptosis‐related lncRNAs was constructed to predict BC prognosis. The risk model showed certain unique features. Firstly, compared with the expression patterns based on all pyroptosis‐related genes, the risk model based on the 8 lncRNAs clearly divided 1,047 BC patients into high‐ and low‐risk groups. Secondly, the risk model had the optimal ability to significantly distinguish the clinical characteristics between high‐ and low‐risk groups, including death probability, clinical stage and TNM stage. Thirdly, the risk score was an excellent independent prognostic factor characterized by good sensitivity and specificity. Moreover, the high‐risk group was also enriched in the biological process associated with tumour malignant progression, supporting the apparent differences between two subgroups divided by the risk model in BC patients. Besides, the analysis of the relationship between immune state and our risk model found that AC004585.1, AL606834.2 and LINC01871 were positively correlated with the memory B cells infiltration. Notably, qRT‐PCR assay validated that AC009119.1 and Z68871.1 were highly expressed in the high‐risk group, which was consistent with our previous bioinformatic results.

Generally speaking, as a specific form of PCD, pyroptosis is characterized by DNA fragmentation, chromatin condensation, cellular swelling with big bubbles and leakage of intracellular content.^20^ Pyroptosis plays double‐faced roles in tumour progression. The bits of pyroptotic cell death in the tumour central hypoxic area can inhibit anti‐tumour immunity and promote tumour development through inducing chronic tumour necrosis.^21^ On the other hand, pyroptosis in the tumour microenvironment can enhance the immune response and impede tumour progression by inducing acute inflammation.^21^ Unfortunately, the interactive patterns of pyroptosis‐related genes in BC and the potential capability in predicting the prognosis of BC patients remain unclear. Numerous studies illustrated the critical role of lncRNAs in inducing tumour progression or suppression, including BC.^22^ Thus, our study constructed a predictive model in BC prognosis based on 8 pyroptosis‐related lncRNAs, that is, AC004585.1, DLGAP1‐AS1, TNFRSF14‐AS1, AL606834.2, Z68871.1, AC009119.1, LINC01871 and AL136368.1.

To date, the role of 5 lncRNAs in our model, including AC004585.1, DLGAP1‐AS1, TNFRSF14‐AS1, Z68871.1 and LINC01871, has been studied in BC and other cancers. For instance, Ye et al. established a risk scoring system based on 12 lncRNAs such as AC004585.1, which effectively predicted the RFS of patients with hepatocellular carcinoma (HCC).^23^ In particular, the higher expression of AC004585.1, the higher risk of HCC recurrence. Interestingly, Z68871.1 was highly expressed in the high‐risk group of BC patients, whether in the risk model based on autophagy‐related lncRNAs^24^ or BCSC‐related lncRNAs.^25^ These findings were consistent with our results in the forest plot that AC004585.1 and Z68871.1 were risk factors of BC prognosis. Meanwhile, Wu et al. screened out LINC01871 and other 4 autophagy‐related lncRNAs to build the prognostic risk model in BC and found that LINC01871 mainly enriched in low‐risk group.^26^ In bladder cancer, the signature consisting of 7 immune‐related lncRNAs, including TNFRSF14‐AS1, was clarified to be a prognostic marker, whereas the TNFRSF14‐AS1 was considered as a protective effector.^27^ Similarly, these reports also supported our conclusions that LINC01871 and TNFRSF14‐AS1 were prone to prolonging the survival time of BC patients. It was noted that few studies explored the prognostic value of DLGAP1‐AS1 in BC, but the dysregulation of DLGAP1‐AS1 had been reported to function as oncogene roles in several tumour types, including glioma,^28^ colorectal cancer,^29^ gastric cancer^30^, ^31^ and HCC.^32^, ^33^ Our study firstly revealed that DLGAP1‐AS1 was highly expressed in the low‐risk group and played a protective role in BC prognosis.

More interestingly, compared with the TNM staging, most of the 8 pyroptosis‐related lncRNAs were more significantly associated with the clinical stage of BC patients, uncovering that the risk model might link to the inherent biological characteristics and heterogeneity of BC. Notably, the results of GSEA illuminated that the significant difference in OS between the high‐risk and low‐risk groups was mainly concentrated in the different pyroptotic and oncogenic statuses induced by the risk model. Obviously, our risk model constructed by 8 lncRNAs represented more significant pyroptosis characteristics and lower immunity than that constructed by pyroptosis‐related genes. Additionally, the in vitro validation elucidated the differential expression features of 8 pyroptosis‐related lncRNAs in BC tissues between high‐ and low‐risk groups, providing reliable biological evidence of our risk model in predicting BC prognosis. Immune infiltration in the tumour microenvironment is a general feature that is dynamically presented in multiple types of cancer. Several extracellular stimuli can coordinate the dynamic interaction between tumour cells and the immune system to accelerate cancer evolution.^34^ The immune system, on the one hand, destroys immunogenic tumour variants to promote the antitumour effect and, on the other hand, shapes tumour immunogenicity to facilitate tumour progression.^34^ Of particular, the density of intratumoural immune infiltration, defined as immunoscore, has been served to determine the poor or favourable prognosis of cancer.^35^ Intriguingly, the immunoscore exhibited a better predictive power in predicting disease‐specific survival and OS, in comparison to the routine TNM system for colorectal cancer stages I, II and III.^36^ Besides, the joint analysis about the differential densities of immune infiltration in the tumour centre and the invasive margin has been demonstrated to effectively predict the prognosis of BC patients with poor clinicopathological parameters.^35^ In 2020, Sui et al. constructed an immune prognostic model based on 6 immune cells which were significantly related to the OS of BC patients, including resting CD4+ T cells, Treg cells, gamma‐delta T cells, activated NK cells, monocytes and M0 macrophages.^37^ Furthermore, the immune cell infiltration‐based immune score model could effectively and efficiently predict the prognosis of BC patients as well as the effect of chemotherapy. Thus, it is worthy and feasible to explore the prognostic value of the immune infiltration alteration in BC progression.

There are several concerns needed to address in this study. Firstly, although the high expression of pyroptosis‐related lncRNAs and mRNAs was partially validated in vitro, the biological function of pyroptosis‐related lncRNAs and the relationship between lncRNAs and immune infiltration are still not yet fully elucidated, and more detailed verification is needed. Secondly, there is a lack of specific mechanisms by which lncRNAs affect memory B cell infiltration. Considering the complexity and heterogeneity of the TME, the factors leading to immune cell infiltration are not limited to pyroptosis, such as chemotherapy resistance, chemokines and cytokines,^38^ which were not taken into consideration in our study. Moreover, the correlation between pyroptosis‐related lncRNAs and immune cell infiltration is still in the preliminary stage and needs further comprehensive investigation. Thirdly, since the risk model was built mainly based on the TCGA database, a larger external clinical cohort is needed to validate the expressions of the pyroptosis‐related lncRNAs and the predictive value of the risk model in practice.

In conclusion, we successfully established an effective predictive BC model based on 8 pyroptosis‐related lncRNAs, including AC004585.1, DLGAP1‐AS1, TNFRSF14‐AS1, AL606834.2, Z68871.1, AC009119.1, LINC01871 and AL136368.1. More intriguingly, the high‐risk BC patients based on the risk score of this model exhibited the presumptive worse clinical outcomes and vice versa. This constructed well‐validated model based on these 8 pyroptosis‐related lncRNAs, which will provide novel insights for BC prognosis recognition.

## CONFLICT OF INTEREST

The authors declare that they have no conflict of interest.

## AUTHOR CONTRIBUTIONS

**Wenchang Lv:** Data curation (lead); Formal analysis (lead); Investigation (lead); Methodology (lead). **Yufang Tan:** Data curation (supporting); Formal analysis (supporting); Investigation (lead); Writing‐original draft (lead); Writing‐review & editing (lead). **Chongru Zhao:** Formal analysis (supporting); Investigation (supporting); Methodology (supporting); Writing‐original draft (supporting). **Yichen Wang:** Data curation (supporting); Formal analysis (supporting); Investigation (supporting); Methodology (supporting). **Min Wu:** Conceptualization (supporting); Project administration (supporting); Supervision (supporting); Validation (supporting). **Yiping Wu:** Funding acquisition (lead); Project administration (lead); Supervision (lead); Validation (lead). **Yuping Ren:** Supervision (lead); Validation (lead). **Qi Zhang:** Supervision (lead); Validation (lead).

## CONSENT FOR PUBLICATION

All authors have provided their consent for publication.

## Supporting information

Tab S1

Tab S2

## Data Availability

All the datasets displayed in this study can be obtained in the online database. Further questions can be directed to the corresponding author.
