# Supplementary material for: Identification of pyroptosis‐related lncRNAs for constructing a prognostic model and their correlation with immune infiltration in breast cancer
Source: J Cell Mol Med. 2021 Oct 10;25(22):10403–17. doi: 10.1111/jcmm.16969 (PMC8581320; doi:10.1111/jcmm.16969)
Supplement: Supplementary file 1 — Tab S1 [file JCMM-25-10403-s001.docx]

**Table S1.** Primer sequences for 8 pyroptosis-related lncRNAs.

| Gene | sense 5’-3’ | antisense 5’-3’ |
| --- | --- | --- |
| AC004585.1 | TCACGGGGACACAGAAGAAA | TGCTATTTCCACCTCCCCTCT |
| DLGAP1-AS1 | TCCACGGATGTAACCACAGC | GCCAACATCAAACACGAGGG |
| TNFRSF14-AS1 | TCCGGGTTTCCTGTAGAAGG | GCAGAAGCTGAGATGGGAACA |
| AL606834.2 | ACAGTCCACACCTGACACCTC | GCATCAGGGACACCATTGGAA |
| Z68871.1 | ACACCAAACCTTGCATCCCT | ATGTCACACGATGTTCAAGCTG |
| AC009119.1 | TGGTTCTAGGTGCTGTGTGGA | CCCTCTGCTTGTCAGGATCAC |
| LINC01871 | AGGGAGGGCAAAGCATTTCA | GCCCAAGTGGTTTGCATCAG |
| AL136368.1 | AAGGTTTGAGGGGATTCGCTG | ACTGCTTAGGCGAACACTCC |
