# Supplementary material for: Identification of pyroptosis‐related lncRNAs for constructing a prognostic model and their correlation with immune infiltration in breast cancer
Source: J Cell Mol Med. 2021 Oct 10;25(22):10403–17. doi: 10.1111/jcmm.16969 (PMC8581320; doi:10.1111/jcmm.16969)
Supplement: Supplementary file 2 — Tab S2 [file JCMM-25-10403-s002.docx]

**Table S2.** Primer sequences for 5 pyroptosis-related mRNAs.

| Gene | sense 5’-3’ | antisense 5’-3’ |
| --- | --- | --- |
| AIM2 | TGGCAAAACGTCTTCAGGAGG | AGCTTGACTTAGTGGCTTTGG |
| CASP1 | TTTCCGCAAGGTTCGATTTTCA | GGCATCTGCGCTCTACCATC |
| CASP4 | TCCGAATATGGAGGCTGGAC | CGTGTGCGGTTGTTTCTCTC |
| IL18 | TCTTCATTGACCAAGGAAATCGG | TCCGGGGTGCATTATCTCTAC |
| NLRP1 | GCAGTGCTAATGCCCTGGAT | GAGCTTGGTAGAGGAGTGAGG |
